# Some oscillatory phenomena of blood glucose regulation: An exploratory pilot study in pigs

**DOI:** 10.1371/journal.pone.0194826

**Published:** 2018-04-02

**Authors:** Nils Kristian Skjaervold, Kathrine Knai, Nicolas Elvemo

**Affiliations:** 1 Department of Circulation and Medical Imaging, Norwegian University of Science and Technology, Trondheim, Norway; 2 Department of Cardiothoracic Anaesthesia and Intensive Care Medicine, Trondheim University Hospital, Trondheim, Norway; 3 Glucoset AS, Trondheim, Norway; INIA, SPAIN

## Abstract

It is well-known that blood glucose oscillates with a period of approximately 15 min (900 s) and exhibits an overall complex behaviour in intact organisms. This complexity is not thoroughly studied, and thus, we aimed to decipher the frequency bands entailed in blood glucose regulation. We explored high-resolution blood glucose time-series sampled using a novel continuous intravascular sensor in four pigs under general anaesthesia for almost 24 hours. In all time series, we found several interesting oscillatory components, especially in the 5000–10000 s, 500–1000 s, and 50–100 s regions (0.0002–0.0001 Hz, 0.002–0.001 Hz, and 0.02–0.01 Hz). The presence of these oscillations is not permanent, as they come and go. This is the first report of glucose oscillations in the 50–100 s range. The origin of these oscillations and their role in overall blood glucose regulation is unknown. Although the sample size is small, we believe this finding is important for our understanding of glucose regulation and perhaps for our understanding of general homeostatic regulation in intact organisms.

## Introduction

A key feature of physiological regulation is the oscillations and pulsations that are apparent in all advanced organisms. These are believed to be of importance for several regulatory processes, and are seen in different organ systems such as the endocrine system, the respiratory system, the circulatory system, the nervous system, and others. The underlying physiological bases for the oscillatory patterns observed in global variables, is believed to be pulsatile and synchronization mechanisms at lower spatial and temporal levels. [1–3]

The pulsatile release of insulin from the beta-cells of the pancreas has been known for several decades and has been examined in both *in vitro* and *in vivo* studies [4,5]. Insulin is released in synchronized bursts with a periodicity of approximately five minutes. The amount of insulin released with each burst is constantly changing depending on the current blood glucose level (BGL). Even in periods of stable BGL, the consecutive bursts are varying, possibly due to the system perturbing itself to fine-tune its regulation. When studying the BGL in intact organisms with massive repetitive measurements, oscillations with a periodicity of approximately fifteen minutes have been found [6]. However, studies of continuous BGL measurements from subcutaneous sensors indicate that a normal BGL entails a complex regulatory pattern [7][8]. Furthermore, this pattern seems to “decomplexify” both as patients develop diabetes mellitus and as a consequence of critical disease [9–12]. This indicates that there could be several distinct oscillatory components in the native BGL regulation that are yet to be discovered.

We have the developed a method to study BGL changes over time in animals with a highly accurate and quickly responding continuous intravascular sensor [13]. In previous studies, we found that this sensor was able to detect these small oscillations in the BGL [14]. Therefore, in this study, we aimed to decipher BGL oscillations in longer time series in intact pigs.

## Materials and methods

### Animals, anaesthesia and study protocol

The study was approved by the Norwegian State Commission for Animal Experimentation (Oslo, Norway). A total of four domestic pigs were used in the studies (22–28 kg), and they were acclimatized and treated in accordance with the European Convention for the Protection of Vertebrate Animals used for Experimental and Other Scientific Purposes. The animals were premedicated with intramuscular diazepam 10 mg and azaperone 400 mg. Anaesthesia was induced through an intravenous access on the external surface of theear with atropine 1.0 mg, fentanyl 8.0 μg/kg, thiopenthal sodium 4.0 mg/kg and ketamine hydrochloride 8.0 mg/kg. Before intubation, 5 ml of 40 mg/ml lidocaine was applied to the larynx. The animals were ventilated in pressure control mode on a ventilator (Dameca, Copenhagen, Denmark) with initial values of FiO_2_ at 0.30, a tidal volume of 10 ml/kg, PEEP at 6 cmH_2_O and respiratory frequency of 18/min adjusted as needed in order to maintain PaCO_2_ at 4.5–5.5 kPa. Anaesthesia was maintained by isoflurane 0.5–1.0%. Based on clinical response this was supplemented with boluses of fentanyl 50 μg/ml as needed. Intravascular volume was maintained by a bolus of acetated Ringer’s solution 10 ml/kg, followed by a continuous infusion of 10 ml/kg/h throughout the experiment. 5000 IU heparin was administered i.v. to prevent clot formation. The animals were kept on the ventilator for almost 24 hours before euthanasia with pentobarbital 100 mg/kg.

After surgical cut-down, the animals were fitted with a central venous line for fluid and medicine administration in their right internal jugular vein and an arterial line in their left carotid artery. Two intravascular glucose sensors (GlucoSet, Trondheim, Norway) were inserted in each superficial femoral artery after surgical cut-down, and connected to the glucose monitor. Details of the glucose sensor with pre-insertion two-point calibration as well as repetitive post-insertion one-point calibrations are described in [13].

### Data handling and analyses

**Fig 1:**
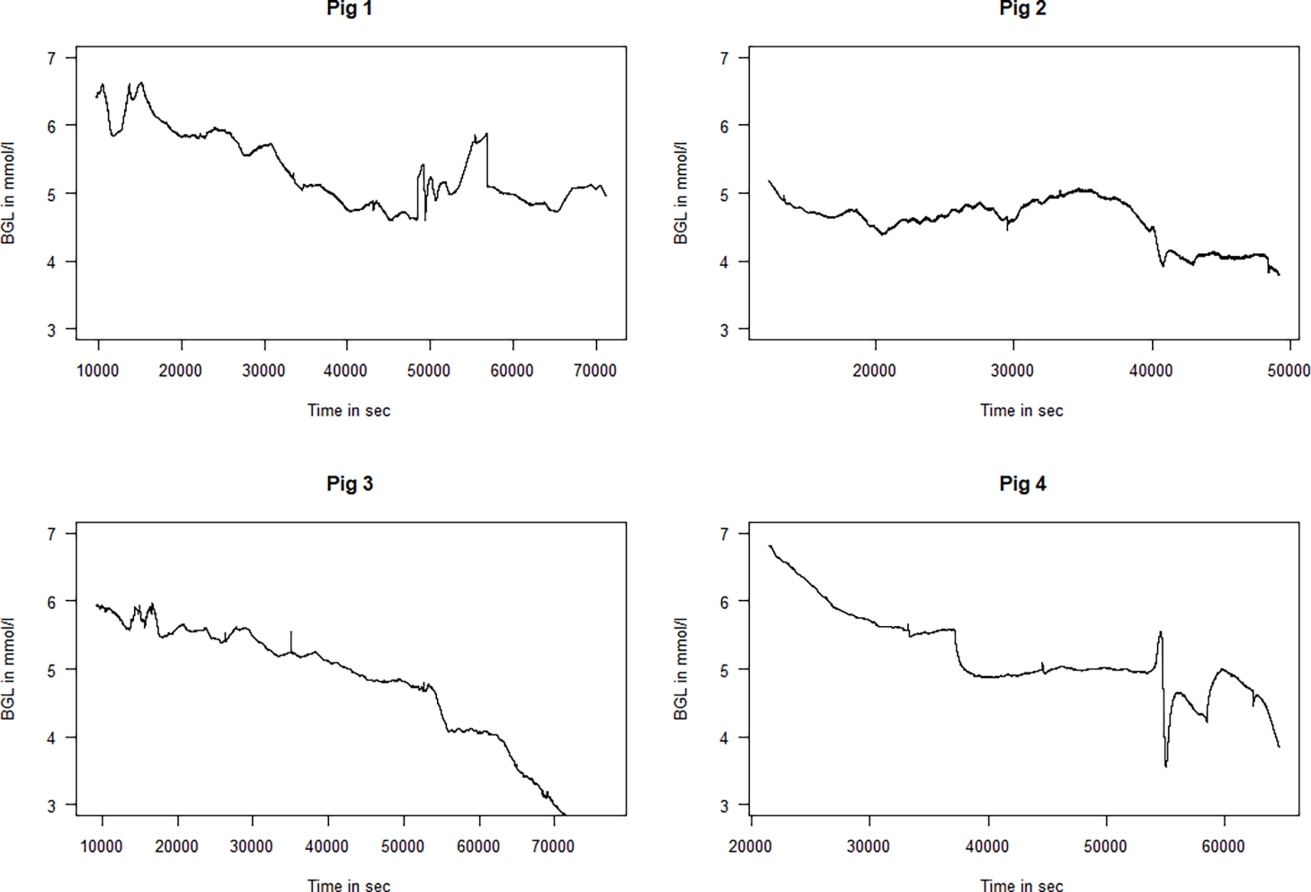
Time series of the glucose values for the four animals. The length of the time series and thus the scaling on the x-axes different between individual animals (BGL = Blood glucose level).

The calibrated glucose signal was exported for analysis with the statistical software “R” version 3.3.0 with the “WaveletComp” package [15,16]. We removed the first 200 min of the sampled data in each series since these were periods of large instability and calibration of the sensors. The rest of the data from the entire study time until sacrifice of the animals are included in the study and are presented in Fig 1. The rest of the data were imported into the statistical software, and a combination of visual inspections and quantitative time-frequency analysis with continuous wavelet analysis was applied, as described in the Results & Discussion section.

The continuous wavelet transform (CWT) is a convolution of the original signal with a function generated from the so-called “mother wavelet” [17]. The mother wavelet is a waveform of limited duration with an average value of zero. In the convolution process, it is shifted in time and stretched and shrunk through the use of a scaling function. By stretching and shifting the mother wavelet in time, the CWT identifies the correlation of different frequencies at different time points. The final wavelet power spectrum is made by making a 3D-display of the correlation values (degree of match = power) and indicates the power by colour.

The most frequently used mother wavelet and the one used in this paper, the Morlet wave, is by mathematical definition a Gaussian enveloped cosine wave [17,18]. To illustrate the oscillatory phenomena, the presence of which varied, we performed the CWT on selections of the time series. Instead of illustrating frequency in Hz (number of cycles per second), we use period (the duration of time of one cycle), specified in seconds.

## Results & discussion

All recordings were mainly performed with BGLs in the range of 4 to 6.5 mmol/l; however, as seen from Fig 1, the BGLs of all animals slowly declined throughout the studies. Some of the BGLs of the animals were very low at the end of the experiment, and the data for these periods were discarded before analysis. When qualitatively studying the BGLs of the four animals (Fig 1) a few oscillatory periods can clearly be seen. In Pig 1 and to some extent in Pig 3, one can see a very slow wave with a period of somewhere between 5000 and 10000 sec (0.0001–0.0002 Hz ≈ 1 ½ h period). In particular, in some parts of Pigs 2, very distinct oscillations with a periodicity of approximately 1000 sec (0.01 Hz ≈ 15 min) can be observed.

**Fig 2:**
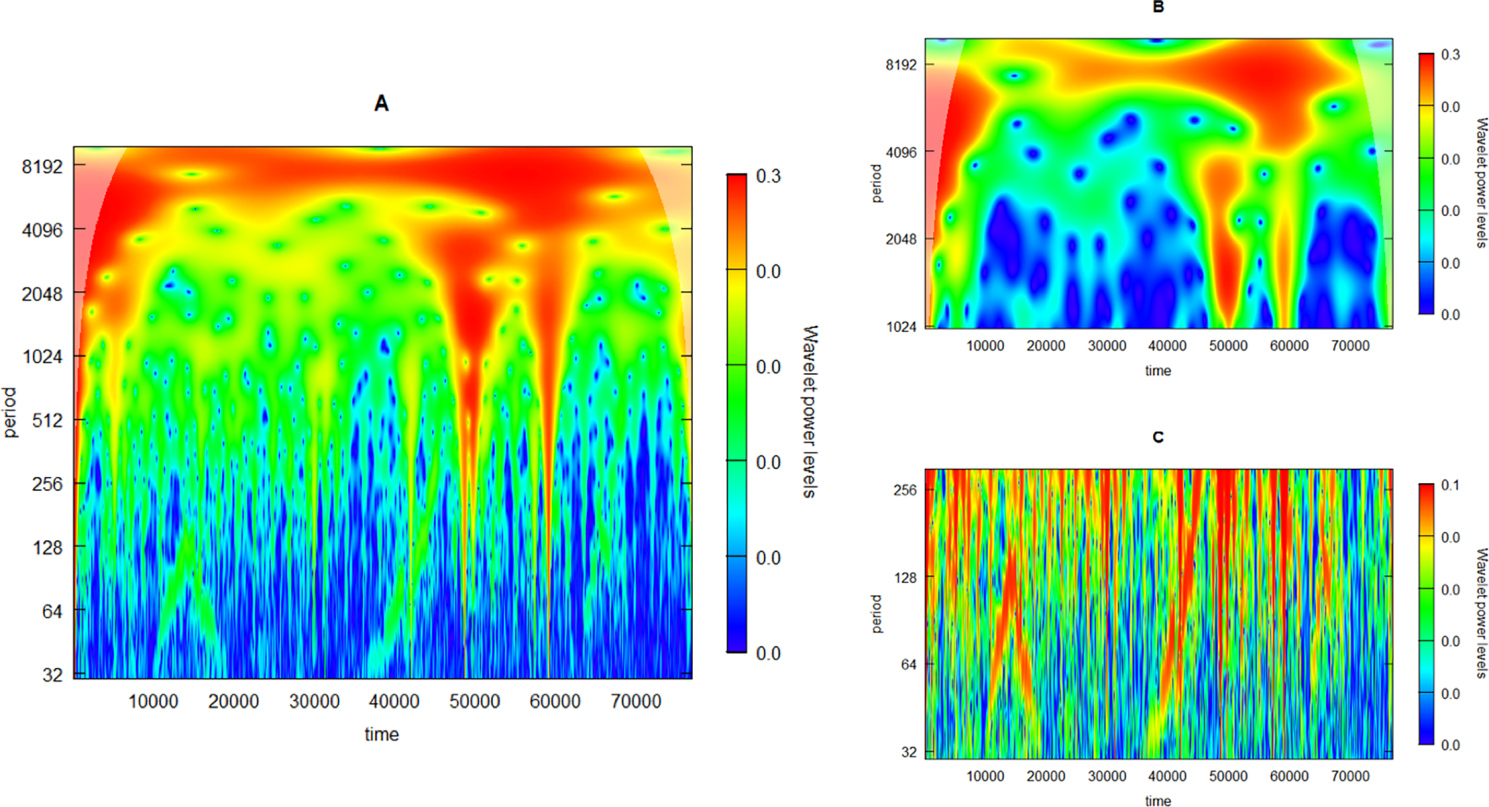
Wavelet power spectrum from the continuous wavelet transform (CWT) of the entire time series from Pig 1. The plot depicts the presence of distinct periods throughout the time series, with the time in the experiment in seconds at the abscissa, the time of the respective periods on a logarithmic scale at the ordinate, and the “power” of distinct periods as a function of the time-series shown in colours according to the scale next to the plots. The CWT covering the whole range of periods from 30–10000 sec only reveals the slow oscillation at approximately 8000 sec (A), which is highlighted when focusing in on the slow periods (B). The CWT of the whole time-series focusing on the high-frequency oscillations at 30–300 sec does not yield any meaningful result (C).

When examining the data in details, as we will below, there are oscillatory components to be found with periodicity ranging from 50 to 5000 sec. These oscillations constitute time-changing properties and should therefore be analysed by a time-frequency method such as the CWT. However, since the oscillatory components are not present throughout the recording and the power of the oscillations compared to the overall signal is so low, it is difficult to merely analyse the signal in its entirety. We subjected the entire time series of Pig 1 to a CWT, looking for periodicity from 30 to 10000 sec, but we had difficulty in deciphering any meaningful information apart from very slow oscillations at a periodicity of approximately 8000 sec (Fig 2A). When repeating the analysis only for the slowest period, we obtained a clearer visualization of this 8000-sec oscillation (Fig 2B). We then repeated the procedure for the shortest periods we studied, 50–100 sec. For these periods, the analysis did not perform well, only indicating some interesting periods in the high frequency range, especially between 10000 and 20000 sec and at 40000 sec in the time series (Fig 2C).

**Fig 3:**
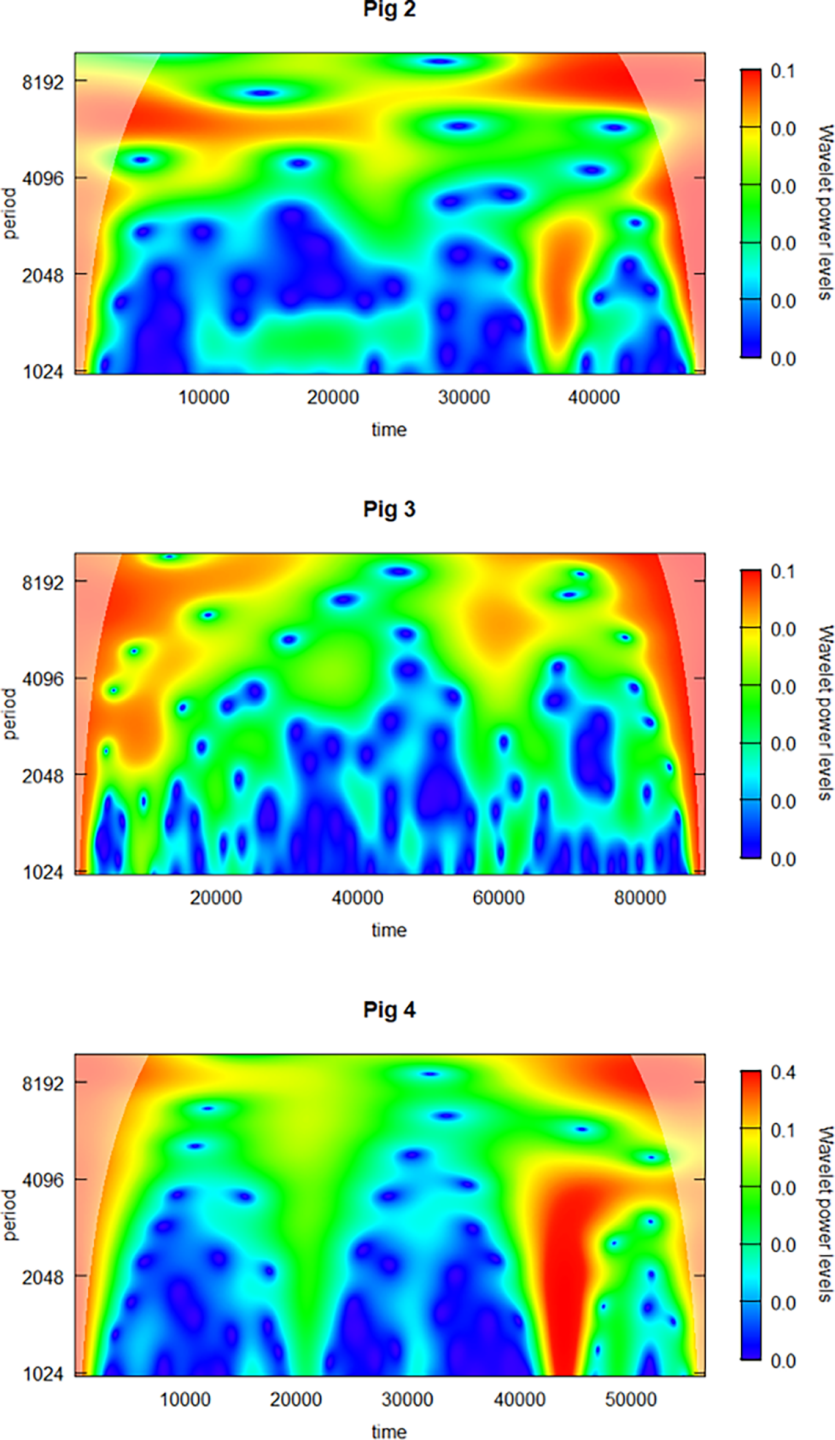
Wavelet power spectrum of the continuous wavelet transform of slow oscillations (1000–10000 seconds) of Pig 2, Pig 3 and Pig 4.

Before going deeper into the faster frequencies, we created a complete time series CWT of the slowest frequencies and performed these analyses for Pigs 2, 3, and 4. Pig 2 also had a low-frequency component at 5000–10000 sec throughout the series, although with larger time-variations than Pig 1. Pig 3 had a very complex oscillatory pattern with an increasing decay in BGL throughout the time-series, that seems to disturb the analysis; however, the 5000–10000-sec oscillatory component can to some extent be seen at the beginning and end of the series. In Pig 4, this component is also present and is most visible in the first and last part of the CWT analysis but less evident in the middle of the analysis period. The lack of such a component in the middle could be caused by several large abrupt changes, artefacts, and the presence of other, more powerful higher-frequency oscillations (Fig 3).

**Fig 4:**
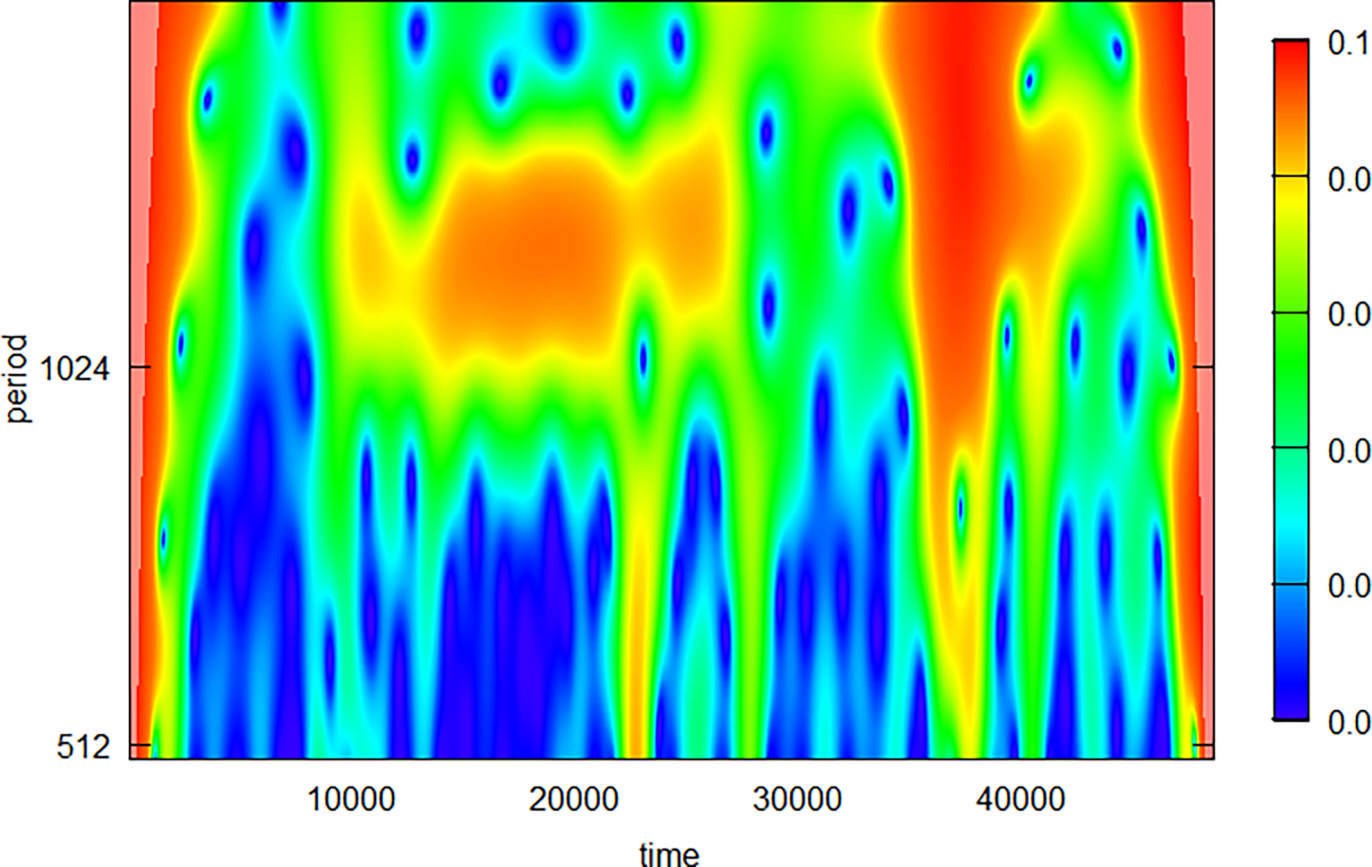
Wavelet power spectrum of the continuous wavelet transform of Pig 2 with a periodicity set to 500–2000 sec to identify the 1000-sec oscillatory component in the second quarter of the time series. This period is somewhat visualized in orange colour in the plot. The high power seen at 35000–40000 sec in the time series is caused by the steep decay in BGL at this time period.

As seen from Fig 1, Pig 2 had very distinct oscillations with a periodicity of some 1000 sec throughout the second quarter of the time series. We therefore specifically searched for this component in this one animal to quantify this component, looking in the range from 500 to 2000 sec. The CWT result is somewhat interesting as it shows both some of the strengths and the weaknesses of the method. One can see the approximately 1000-sec oscillation between 1000 and 3000 sec in the time series, but it does not stand out as very powerful and clear (Fig 4). This is probably caused by a combination of edge effects, artefacts, and other oscillatory components somewhat overshadowing the period we are examining.

**Fig 5:**
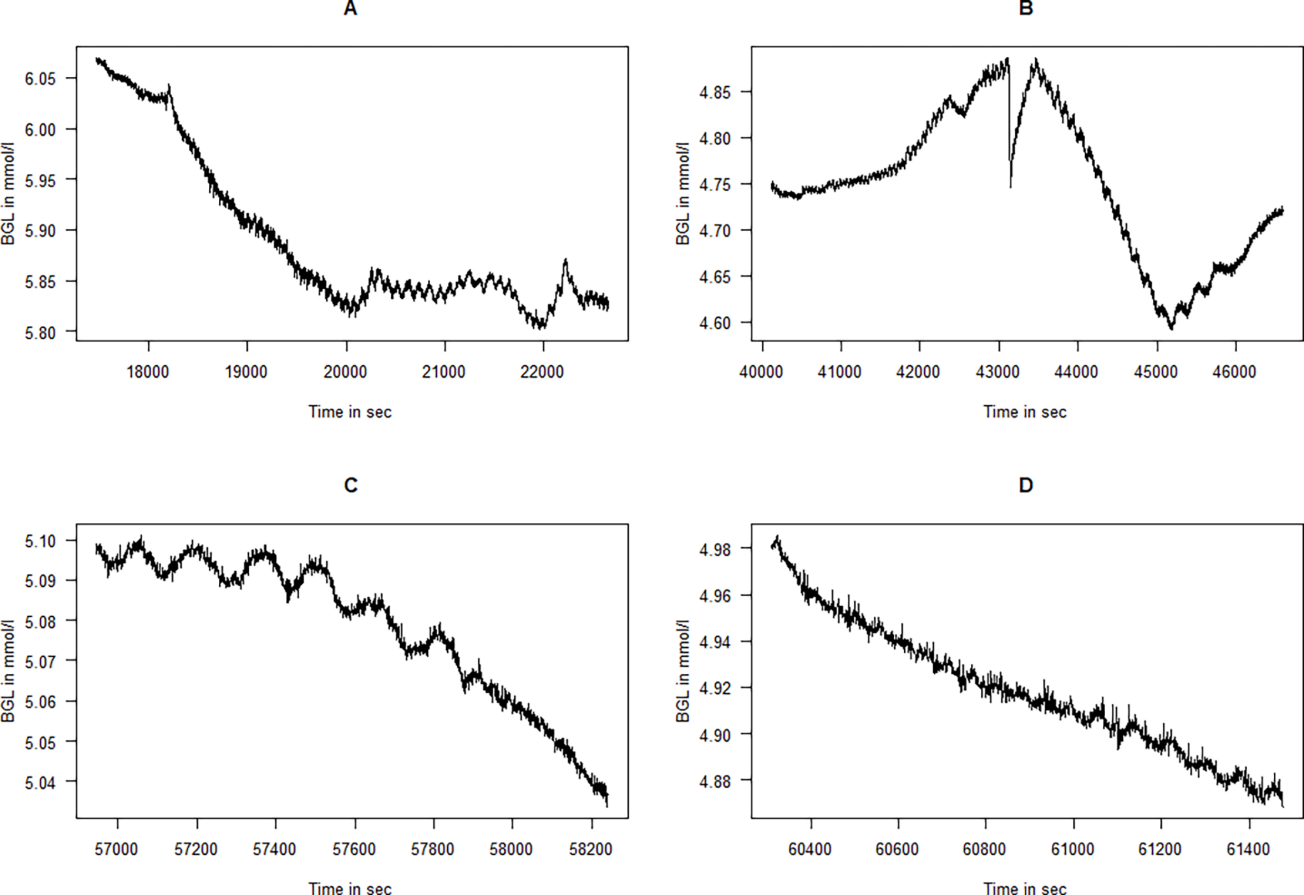
Some interesting periods from Pig 1 with oscillations in the 50–100 sec period range.

**Fig 6:**
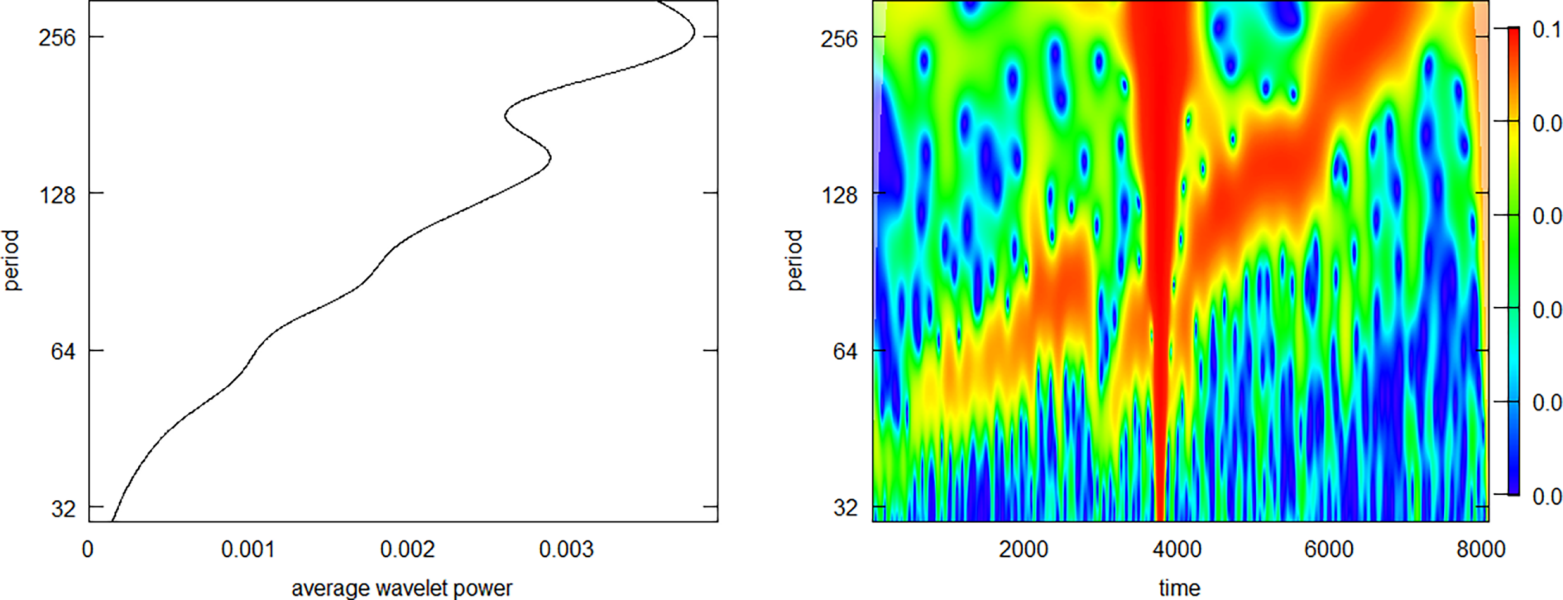
Continuous wavelet transform of the time series from Fig 5B where the periodicity appears to be constantly changing. The average power plot to the left only indicates that there are some periodicities in the 100–300 sec range while the wavelet power spectrum plot to the right clearly shows how the main oscillatory component has a linear rising periodicity from 50 to 300 sec throughout the time series. The abrupt drop in the middle of the series, seen in Fig 5B, yields the large artefact in the middle of the wavelet power spectrum plot.

When looking into the individual time series in more detail, especially searching for faster oscillatory periods, some interesting features appear. Pig 1 has quite a few periods with distinct oscillations in the 50–100 sec range, and Fig 5 depicts some of the most impressive periods. As shown, the exact periodicity varies some, and sometimes it changes in a linear fashion within small time periods; for example, in Fig 5 panel B, there is a quickly oscillating component that seems to have a linear increase in periodicity. Fig 6 depicts a CWT of this individual time period. Here, we have both an average power spectrum that does not consider the time dimension and therefore does not yield much information (left panel), and a time-frequency plot where the oscillatory component is very visible. The latter plot clearly shows how the oscillatory component linearly increases its period from 50 to 300 sec.

**Fig 7:**
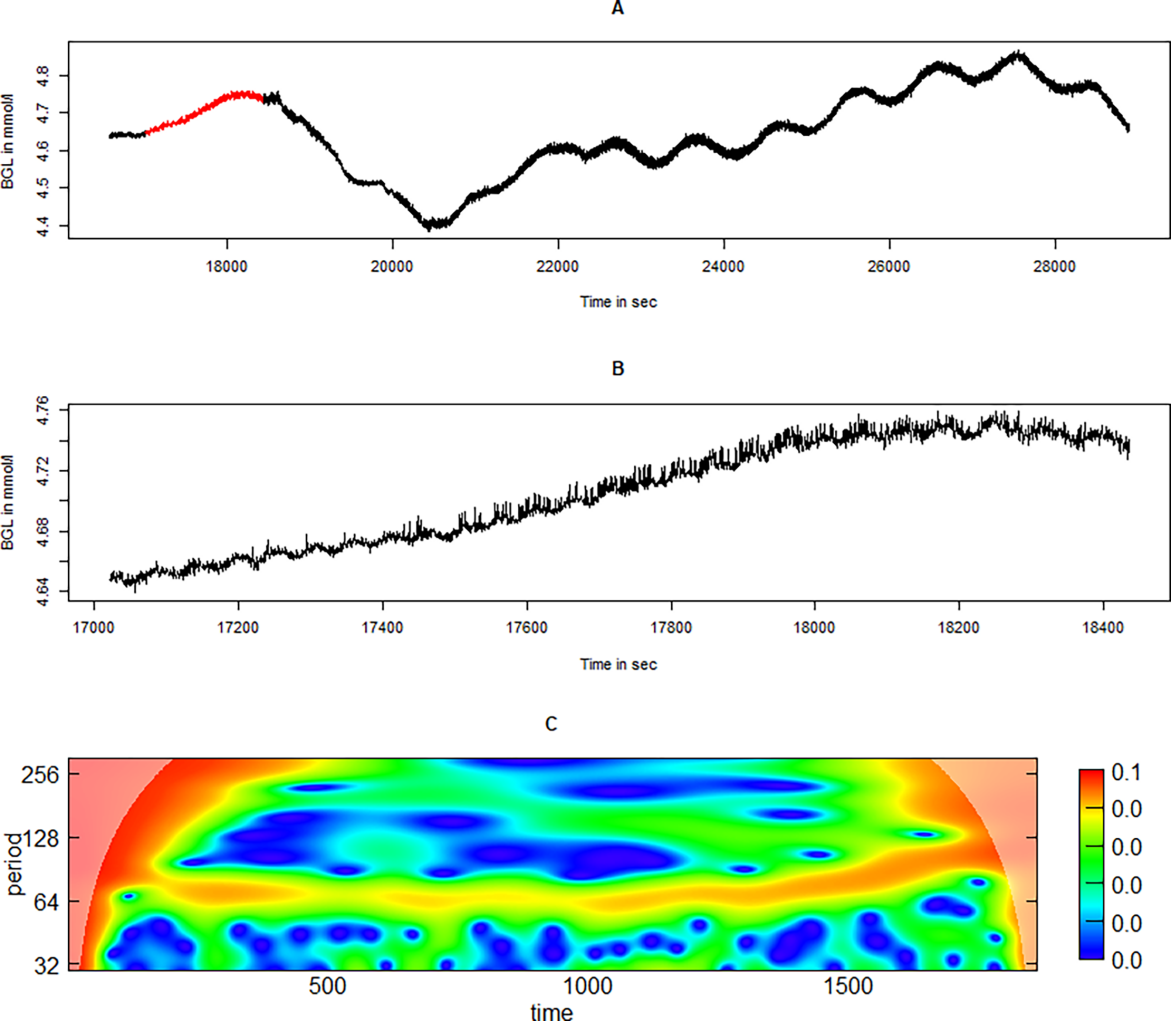
A period from Pig 2 in which two different oscillatory components follows each other. In Fig 7A, the 500–1000-sec slow oscillating component in the second half of the time series is shown. A period (marked in red, enlarged in panel B) seems to have a fast oscillating component at 50–100 sec. The wavelet power spectrum from the continuous wavelet transform of the time series in 7 B is displayed in 7 C, and this clearly shows the 50–100 sec oscillatory component.

Often, one oscillatory component precedes another, or different oscillatory components are present at the same time in a fractal-like pattern. For example, in Pig 2, there is a distinct period of 50–100-sec oscillations followed by the very characteristic 1000 sec oscillation (Fig 7)

**Fig 8:**
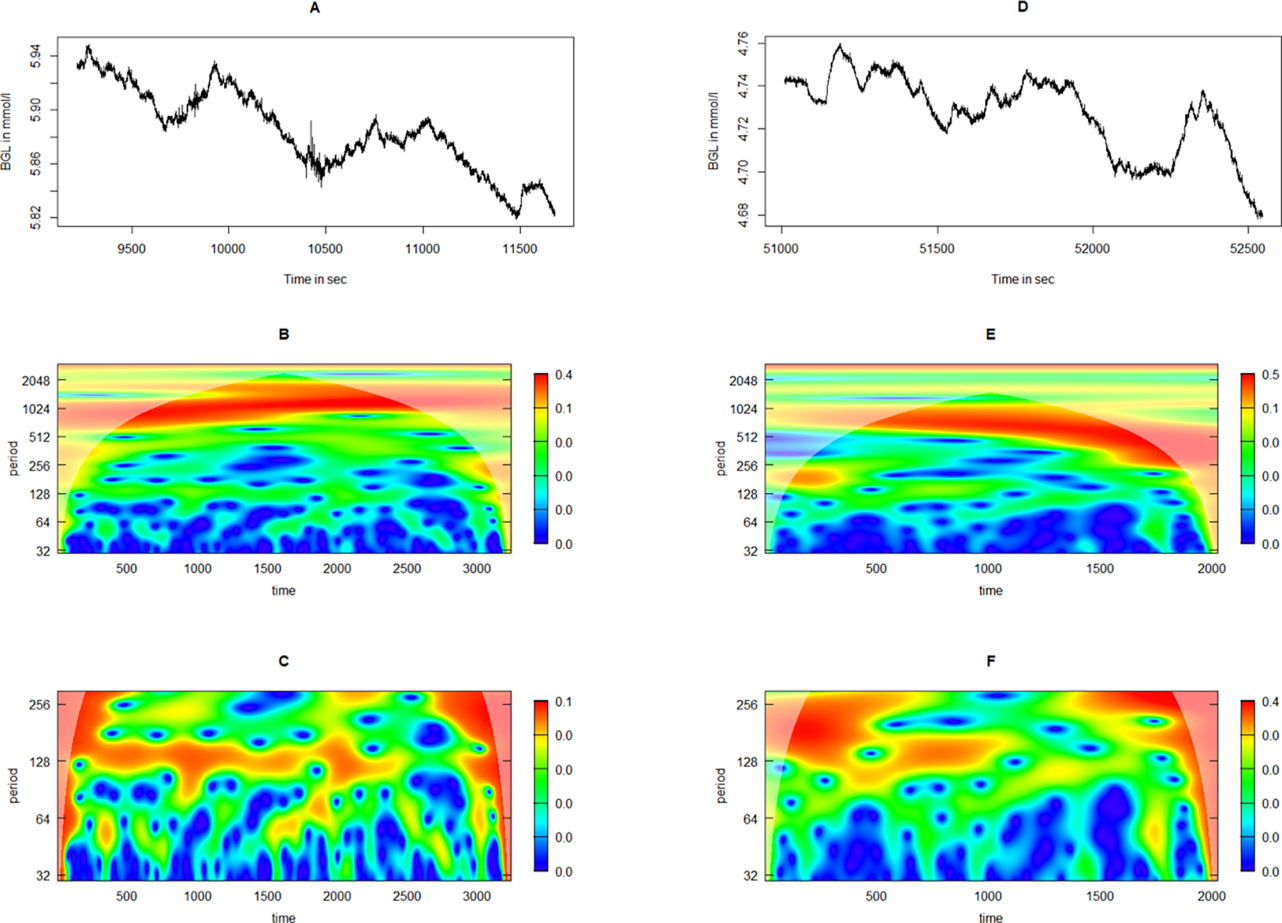
The fractal nature of blood glucose oscillations illustrated with two examples from Pig 3. Panels A and D depicts the time series from two situations in which the 1000-sec oscillation is clearly seen. Panels B and E depicts the wavelet power spectrum from the continuous wavelet transform from A and D, respectively, clearly showing the 1000-sec oscillatory component. However, the 50–100-sec component is poorly depicted in these figures due to the low power in the high-frequency oscillations compared with the low-frequency oscillations. Thus, panels C and F depicts the 50–100-sec components from A and D, respectively. (BGL = blood glucose level).

However, the fractal nature is most clearly seen in Pig 3, where Fig 8 depicts two illustrative situations in which both the 100-sec and the-1000 sec oscillations are present at the same time.

**Fig 9:**
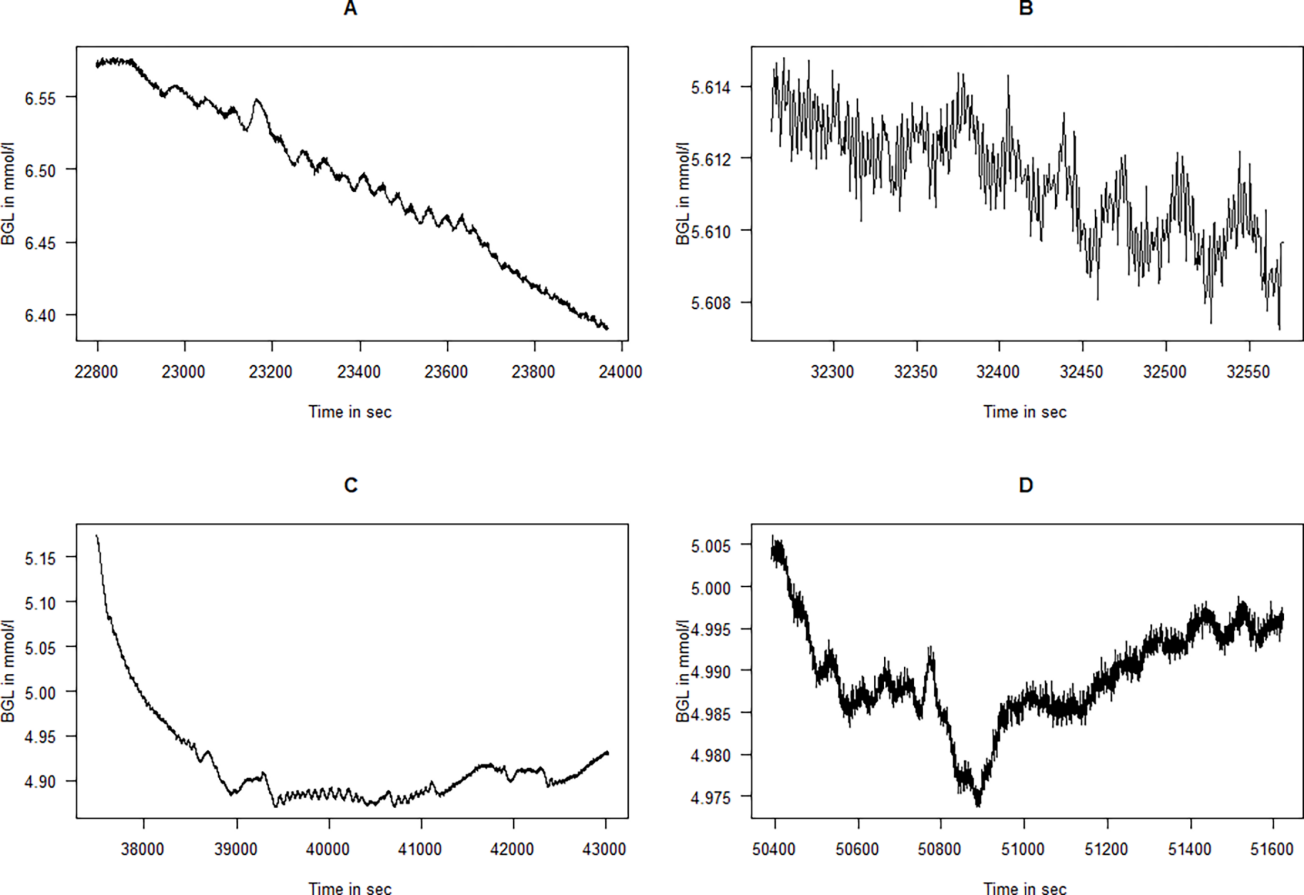
Some interesting periods from Pig 4 with oscillations in the 50–100-sec range and the 1000-sec range.

In Pig 4, we found several oscillatory components, especially in the 50–100-sec range and the 1000-sec region, as shown in Fig 9.

The current study was exploratory in its nature, and should be interpreted as hypothesis generating. We also kept in mind that we studied pigs, not humans, and the very limited number of animals observed. The results of this pilot animal study need to be confirmed in larger future studies, preferably in humans, to draw clearer conclusions. Nevertheless, these examples show several oscillatory components in the time series of all four pigs. In our study, we find clear oscillations in the 5000–10000-sec, 500–1000-sec, and 50–100-sec regions (0.0002–0.0001 Hz, 0.002–0.001 Hz, and 0.02–0.01 Hz). This could indicate that there are three distinct oscillators within the organism that regulate the BGL. It is beyond the scope of this exploratory study to speculate regarding the physiological origin of these oscillations. However, based on our previous studies on the effect of intravenous insulin boluses on BGL changes in pigs, where the effect of each bolus has an approximately 15-min BGL-lowering effect [19], the fastest oscillations are unlikely to be caused by the pulsatile oscillation release by the beta-cells. The two slowest effects, in contrast, could be caused by such pulsatility.

The current study confirms observations of previously described oscillations using a novel sensing system, and describes a previously undescribed high-frequency oscillatiory phenomenon. There have been some concerns that the observed differences in complexity in continuous glucose measurements in different clinical situations could be caused by limitations in the sensors [20]. However, the novel sensing system used in this study has a very low signal-to-noise ratio, as seen in the figures containing unprocessed raw data (Figs 1, 5, 7 and 8). The high-frequency phenomenon is unlikely to have been picked up with slower glucose sensors. Nevertheless, the sensors used in this study have a time constant to stepwise change of some two minutes. The amplitudes of the measured oscillations are therefore likely larger than those that we measure in these experiments due to damping caused by this time-delay.

A limitation of using a novel sensing system is that it is not as well-described as a more mature system and that the results could be caused by an unknown interference or other phenomenon in the sensing system. However, the non-stationarity and physiological appearance of the oscillations make them unlikely to be caused from some unknown properties of the optical signal processing. Prior to this study, the sensors were tested in *in vitro* studies to examine interference by other physiological and pharmacological chemical factorss, and they were found to be sensitive to interferents, mainly temperature and pH. However, while these measures can change quickly in experimental conditions [21], no changes in the experimental protocol varied with the same frequency as the oscillations. Alternatively, could the observed BGL oscillations be caused by some other natural oscillatory phenomena or iatrogenic interference from medications or other interventions? All medication given throughout a study can theoretically have some unappreciated effect on the physiological outcome studied [22], for example, isoflurane is known to interfere with the insulin/glucose system, albeit not in an oscillatory manner [23]. It is also unlikely that the oscillatory nature of the respirator with a periodicity of three to four seconds or occasional changes in anaesthesia could cause the rhythmicity observed in the BGL. Nevertheless, the current study cannot rule out that the observed oscillations were in fact caused by some previously undescribed non-glucose, high-frequency phenomenon.

## Conclusion

In this exploratory study of continuous intraarterial BGL measurements in four domestic pigs under general anaesthesia, we found several interesting oscillatory components, especially in the 5000–10000-sec, 500–1000-sec, and 50–100-sec regions (0.0002–0.0001 Hz, 0.002–0.001 Hz, and 0.02–0.01 Hz). The origin of these oscillations is unknown. Further studies are needed to confirm the novel findings described in this study and to elucidate any underlying physiological mechanism of the phenomena.

## Supporting information

S1 Text A table of time (sec) in column 1 and blood glucose values (mmol/l) in column 2 from the entire recording in Pig 1. (TXT)

S2 Text A table of time (sec) in column 1 and blood glucose values (mmol/l) in column 2 from the entire recording in Pig 2. (TXT)

S3 Text A table of time (sec) in column 1 and blood glucose values (mmol/l) in column 2 from the entire recording in Pig 3. (TXT)

S4 Text A table of time (sec) in column 1 and blood glucose values (mmol/l) in column 2 from the entire recording in Pig 4. (TXT)
